# The Efficacy of the BCG Vaccine against Newly Emerging Clinical Strains of *Mycobacterium tuberculosis*

**DOI:** 10.1371/journal.pone.0136500

**Published:** 2015-09-14

**Authors:** Marcela Henao-Tamayo, Crystal A. Shanley, Deepshikha Verma, Andrew Zilavy, Margaret C. Stapleton, Synthia K. Furney, Brendan Podell, Ian M. Orme

**Affiliations:** Mycobacteria Research Laboratories, Department of Microbiology, Immunology and Pathology, Colorado State University, Fort Collins, Colorado, United States of America; University of Cape Town, SOUTH AFRICA

## Abstract

To date, most new vaccines against *Mycobacterium tuberculosis*, including new recombinant versions of the current BCG vaccine, have usually been screened against the laboratory strains H37Rv or Erdman. In this study we took advantage of our recent work in characterizing an increasingly large panel of newly emerging clinical isolates [from the United States or from the Western Cape region of South Africa], to determine to what extent vaccines would protect against these [mostly high virulence] strains. We show here that both BCG Pasteur and recombinant BCG Aeras-422 [used here as a good example of the new generation BCG vaccines] protected well in both mouse and guinea pig low dose aerosol infection models against the majority of clinical isolates tested. However, Aeras-422 was not effective in a long term survival assay compared to BCG Pasteur. Protection was very strongly expressed against all of the Western Cape strains tested, reinforcing our viewpoint that any attempt at boosting BCG would be very difficult to achieve statistically. This observation is discussed in the context of the growing argument made by others that the failure of a recent vaccine trial disqualifies the further use of animal models to predict vaccine efficacy. This viewpoint is in our opinion completely erroneous, and that it is the fitness of prevalent strains in the trial site area that is the centrally important factor, an issue that is not being addressed by the field.

## Introduction

Tuberculosis remains a global emergency, with ~9-million new cases occurring each year, and 1.5-million deaths [1]. The incidence of new infections that are drug-resistant is now estimated at nearly half a million cases [2], leading increasingly to poor treatment outcomes and increases in mortality. Much of the current epidemic is driven by the concomitant HIV epidemic [3], particularly in Southern Africa, and other risks factors are also emerging, including diabetes [4,5]. As a result, considerable effort is being made to try to develop new vaccines and drugs to combat the tuberculosis epidemic.

A renewed effort to develop new vaccines—either to improve the existing BCG vaccine or to replace or boost it—began in earnest 25-years ago, but unfortunately progress has been slow, and the first Phase IIb efficacy study was only recently completed. In that study [6] a virus-delivered vaccine [MVA85A] was tested for its ability to boost BCG vaccination in infants [4–6 months of age], which it failed to do so. This outcome has led to a re-evaluation of vaccine development, including a discussion as to whether animal models should still be used to screen vaccines pre-clinically since [in the case of MVA85A] they were not predictive. This viewpoint is erroneous, as will be discussed below.

As recently discussed [7] one possible limitation in the current field is the reliance on laboratory-adapted strains of *M*.*tuberculosis* [H37Rv, Erdman] to screen vaccines. This is an important point, because studies of newly emerging strains indicate that they express a much wider range of virulence and fitness, and often as not a broader range of T cell subset responses [8–10]. In addition, because the “window of protection” that occurs in BCG vaccinated mice challenged with H37Rv/Erdman is relatively modest, it is possible to demonstrate the effect of boosting vaccines in this type of model if these strains are used.

In the current study we addressed the question of whether BCG is equally effective against newly emerging clinical strains of *M*.*tuberculosis* in mouse and guinea pig models—the two most widely used animal screens. In some experiments, in addition, we also included a newly developed recombinant BCG vaccine candidate, Aeras-422, a BCG Danish strain which over-expresses Ag85A and Ag85B, Rv3407, and a mutant form of the perfringolysin gene. Although this candidate triggered a safety signal early during clinical trials which precluded its further progress [11] it nevertheless provides an excellent example of the new generation rBCG candidates.

The results of this study further revealed various outcomes that can occur in these types of models, in that BCG is protective against the majority of strains tested, but poorly or transiently protective against certain others. This was predominantly seen in the case of strains obtained from around the United States, whereas BCG was consistently highly protective against all strains tested from the Western Cape region of South Africa. In addition, however, while the primary concept behind new rBCG vaccines is better immunogenicity and protection, we did not observe this in our current studies, and in fact in long term survival studies BCG was far more protective than rBCG.

These results indicate that BCG can give rise to a range of protective efficacy against different clinical isolates. This is not directly related to virulence, since all the isolates used here grew well in the animal models, but instead seems to point to bacterial fitness as a major factor. In this regard, if, as these results suggest, Western Cape strains are generally of low fitness [spreading as they do in a region where malnutrition and high rates of HIV are major factors] and as a result are highly inhibited by prior BCG vaccination as shown below, then it would be very difficult if not impossible to demonstrate in these models any positive effects of boosting regimens. We will discuss these results in the direct context of the MVA85A trial, the result of which was directly predicted in retrospect by the animal models used here.

## Materials and Methods

### Animals

Specific-pathogen-free female C57BL/6 mice, 6 to 8 weeks old, were purchased from the Jackson Laboratories (Bar Harbor, ME). Mice were maintained in the biosafety level III facilities at Colorado State University and were given sterile water, chow, bedding, and enrichment for the duration of the experiments. The specific-pathogen-free nature of the mouse colonies was demonstrated by testing sentinel animals. All experimental protocols were approved by the Animal Care and Use Committee of Colorado State University.

Specific pathogen free, female outbred Hartley guinea pigs (∼450–500g in weight) were purchased from the Charles River Laboratories (North Wilmington, MA) and held under barrier conditions in a biosafety level III animal laboratory. The specific-pathogen-free nature of the guinea pig colonies was demonstrated by testing sentinel animals. All experimental protocols were approved by the Animal Care and Usage Committee of Colorado State University and comply with NIH guidelines. Prior to *M*. *tuberculosis* challenge, animals were appropriately acclimatized, then microchipped for individual animal identification.

### Experimental infections

*M*.*tuberculosis* H37Rv was originally obtained from the Trudeau Institute [NY] collection. Three strains were collected in the Bay Area of California and kindly provided by Dr. Midori Kato-Maeda [University of California, San Francisco]; these were the Beijing strains 4619, 3446, and 3507. Five strains were collected in the Western Cape region of South Africa and kindly provided by Dr. Tommie Victor and Dr. Elizabeth Streicher; four of these strains are Beijing strains [954, 212, R3180, and 3382] while a fifth, 923, is a Haarlem family strain. All strains were grown in 7H9 broth containing 0.05% Tween 80, OADC, and glycerol. When cultures reached an OD_600_ reading of 0.600–1.00 they were bottled, frozen, and then titered.

Mice were infected using a Glas-Col aerosol generator (Glas-Col, Terre Haute, IN), calibrated to deliver 50–100 bacteria into the lungs. A Madison chamber aerosol generation device was used to expose guinea pigs to the different strains of *M*.*tuberculosis*. This device was calibrated to deliver approximately 10–20 bacilli into the lungs. Thawed aliquots of frozen cultures were diluted in sterile saline to the desired inoculum concentrations. The infection inoculum and was determined for all the bacterial strains tested by plating serial dilutions of inoculum on nutrient 7H11 agar and counting CFU three weeks later. No significant differences in terms of the infection dose were seen among any of the strains tested.

Bacterial loads were determined by plating serial dilutions of individual whole organ homogenates on nutrient 7H11 agar. CFU were counted after incubation for 3 weeks at 37°C in humidified air.

### Histological analysis

The right caudal lung lobe from each mouse was fixed with 4% paraformaldehyde in phosphate-buffered saline. Sections from these tissues were stained with hematoxylin and eosin. Similarly, lung lobes from each guinea pig were fixed and stained with hematoxylin and eosin and evaulated by a veterinary pathologist.

### Vaccinations

Animals were vaccinated with BCG Pasteur or with recombinant BCG Aeras-422 [a kind gift of the Aeras Foundation]. Mice were vaccinated with 1x10^6^ bacilli by the subcutaneous route. Guinea pigs were vaccinated with 1x10^4^ bacilli by the intradermal route.

### Flow cytometry

Mice were euthanized by CO2 asphyxiation, and the thoracic cavity was opened. The lung was cleared of blood by perfusion through the pulmonary artery with 10 ml of ice-cold phosphate buffered saline (PBS) containing 50 U/ml of heparin (Sigma, St. Louis, MO). Lungs were aseptically removed, teased apart and treated with a solution of DNase IV (DNase) (Sigma Chemical; 30 μg/ml) and collagenase XI (Sigma Chemical; 0.7 mg/ml) for 30 min at 37°C. Erythrocytes were lysed with Gey's solution (0.15 M NH4Cl, 10 mM HCO3), and the cells were washed with Dulbecco's modified Eagle's minimal essential medium. Total cell numbers were determined by flow cytometry using BD liquid counting beads, as described by the manufacturer (BD Pharmingen, San Jose, CA).

Single-cell suspensions of lung from each mice were resuspended in PBS (Sigma-Aldrich) containing 0.1% of sodium azide, and 4% BSA. Fc receptors were blocked with purified anti-mouse CD16/32. Cells were incubated in the dark for 25 min at 4°C with predetermined optimal titrations of specific antibodies. Cell surface expression was analyzed for CD4 and CD8. Antibodies were purchased from BD Pharmingen. Samples were analyzed on a Becton Dickinson LSR-II instrument, and data was analyzed using FACSDiva v7.0 software. Individual cell populations were identified according to the presence of specific fluorescence-labeled antibodies. All the analyses were performed with acquisition of a minimum of 300,000 events.

To detect IFN-g-positive lymphocytes, *c*ells were initially stimulated for 4hr at 37°C with 1X cell stimulation cocktail (eBioscience) diluted in complete DMEM. Thereafter, cells were stained for cell surface markers as indicated above, then fixed and permeabilized using a Fix/Perm and Perm wash kit (eBioscience). Thereafter, cells were incubated for 30 min at 4°C with FcBlock plus anti-IFN- g (clone XMG1.2, eBioscience), or with the respective isotype control. Data acquisition and analysis was performed as described above.

### Kaplan Meier analysis

The ability of the vaccines to provide long term protection was tested using the Western Cape strains 3382 and the drug-resistant strain R3180. The survival of the animals was monitored by weighing and observation based on a modified Karnofsky scale. A guinea pig was euthanized if the animal showed extensive labored breathing, was lethargic, had a matted or scruffy coat, dark eye color, non-responsive and/or if the weight loss was greater than 20% of the weight of the animal recorded at the time of challenge.

## Results

### Efficacy studies in the mouse model

**Fig 1:**
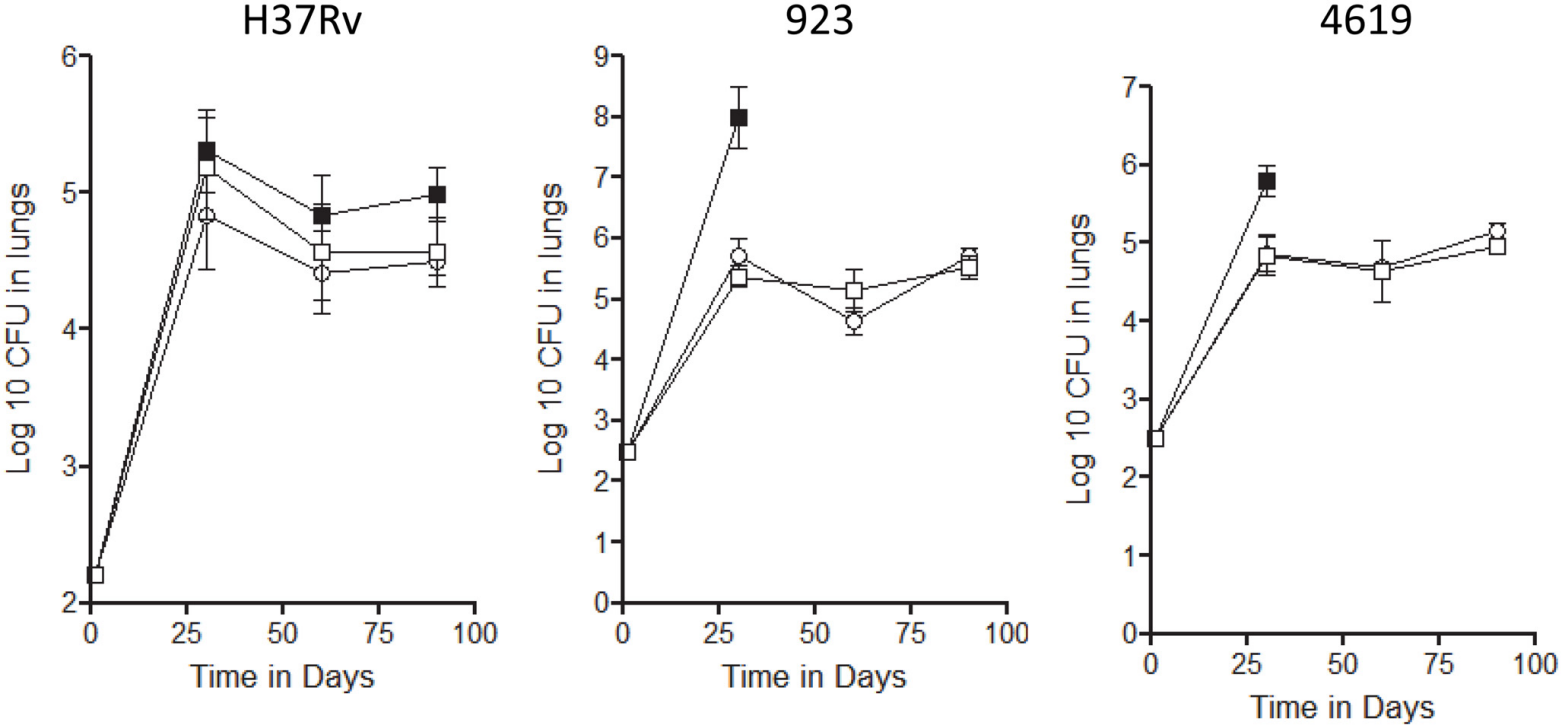
Course of infection in C57BL/6 mice infected by low dose aerosol exposure to the laboratory strain H37Rv, Haarlem strain 923, or Beijing strain 4619. Calculated CFU are shown against time for saline controls [closed squares] and in mice previously vaccinated subcutaneously with BCG Pasteur [open circles] or rBCG Aeras 422 [open squares]. Data is shown for groups of five mice ± SEM. Given the morbidity and mortality seen in control groups infected with the two clinical strains only the day-30 data is reliable.

The effects of BCG and the rBCG were tested in the standard low dose aerosol infection model in the mouse (Fig 1) against H37Rv and two clinical strains, Haarlem-923 [from the Western Cape] and Beijing-4619 [from the Bay Area, California]. Both BCG and rBCG slowed the progression of H37Rv, but because the unvaccinated control mice themselves reduced the bacterial load in the lungs it was not until day-90 that this reached a statistical level [P = 0.045]. In the case of the two clinical strains, both rapidly killed infected mice in 60–70 days [only the day-30 data is reliable as a consequence]. In the case of vaccinated animals however both sets of animals were highly protected by vaccination with the two clinical isolates both halted after reaching ~5-log in the lungs.

**Fig 2:**
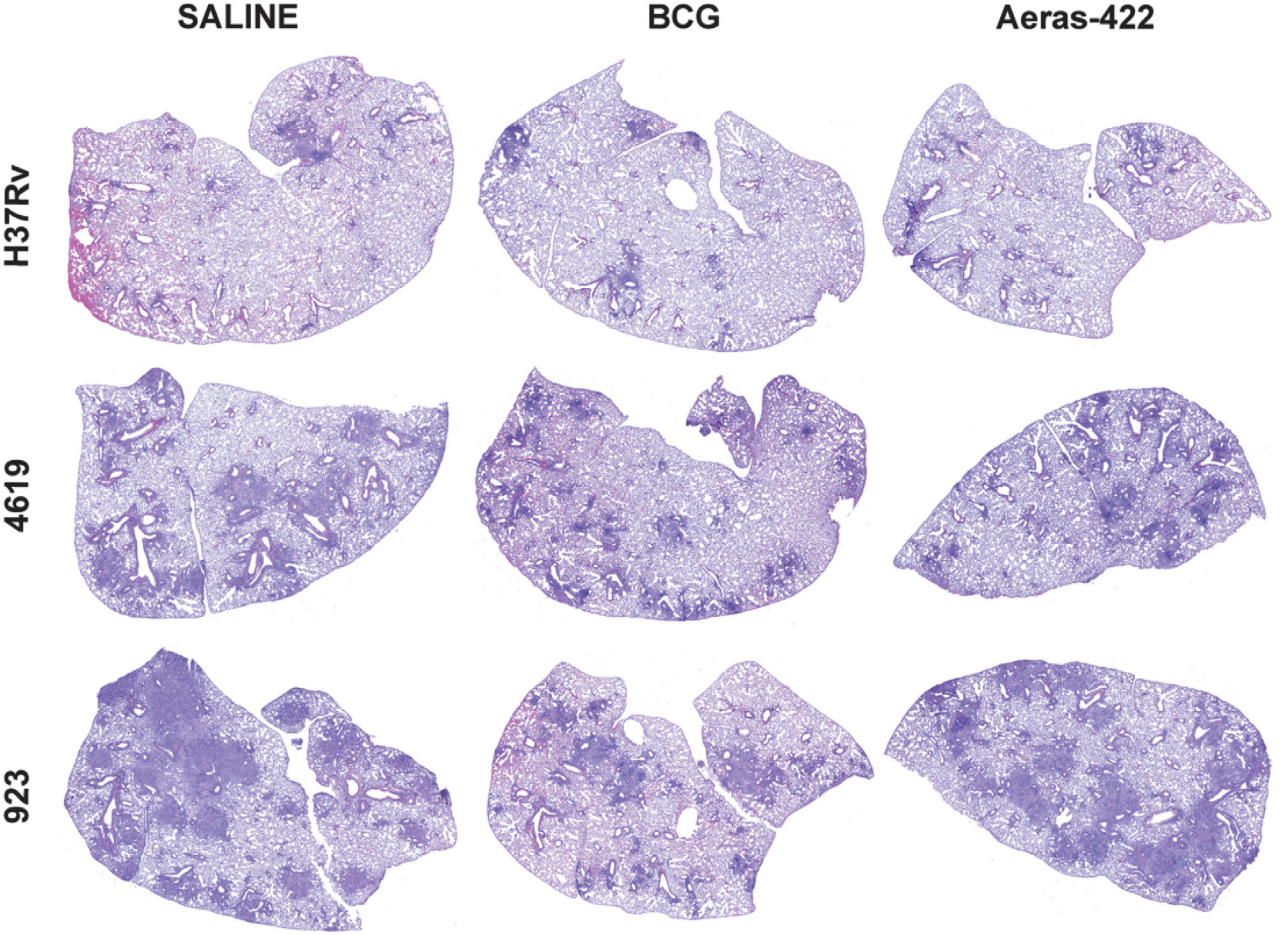
Representative histology images demonstrating overall disease burden in mice infected with H37Rv, Beijing 4619, or Haarlem 923, and either sham-immunized or immunized with BCG or 422. Mild disease burden is present mice infected with H37Rv, with minimal differences in mice immunized with either BCG or 422. Progressive pulmonary pathology developed in unprotected mice infected with clinical strains, 4619 or 923, with most severe disease occurring in mice infected with 923. Protection based on overall lesion burden is afforded by both BCG and 422 against infection with the two clinical strains. However, in mice infected with the 923 strain, more severe pathology developed in mice immunized with 422 compared to BCG.

**Fig 3:**
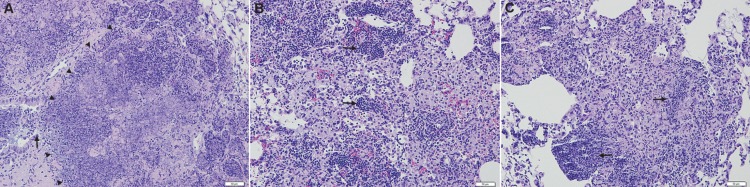
Representative histology images demonstrating leukocyte composition and necrosis in 923-infected mice. [A] Large areas of pulmonary necrosis (arrowheads) developed in mice sham-vaccinated and infected with the 923 clinical strain, consisting of high numbers of degenerate neutrophils with destruction of adjacent airways (arrow). [B] Protection from progressive pulmonary pathology was afforded by BCG vaccination, with development of lymphocyte-rich lesions (arrows) and a complete absence of necrosis. [C] Neutrophil-dominated inflammation and necrosis in a mouse immunized with rBCG 422 (arrows). The degree of inflammation is less severe than in sham-immunized controls.

Consistent with these results, vaccination had a protective effect in terms of lung pathology in all three cases [the 4619 study is shown as an example in Fig 2]. In control animals infected with 4619 the lesions had large numbers of neutrophils present and extensive disease affecting up to 60% of the lung area. Small areas of necrosis could be seen. In the vaccinated mice granulomas consisted predominantly of macrophages with fewer lymphocytes, with the lung burden and cellular composition reduced to about 25–40% in mice vaccinated with BCG. About 40% of the lung tissue in mice vaccinated with Aeras-422 was affected, and small areas of necrosis could be seen (Fig 3).

### T cell subset profiles in vaccinated mice

**Fig 4:**
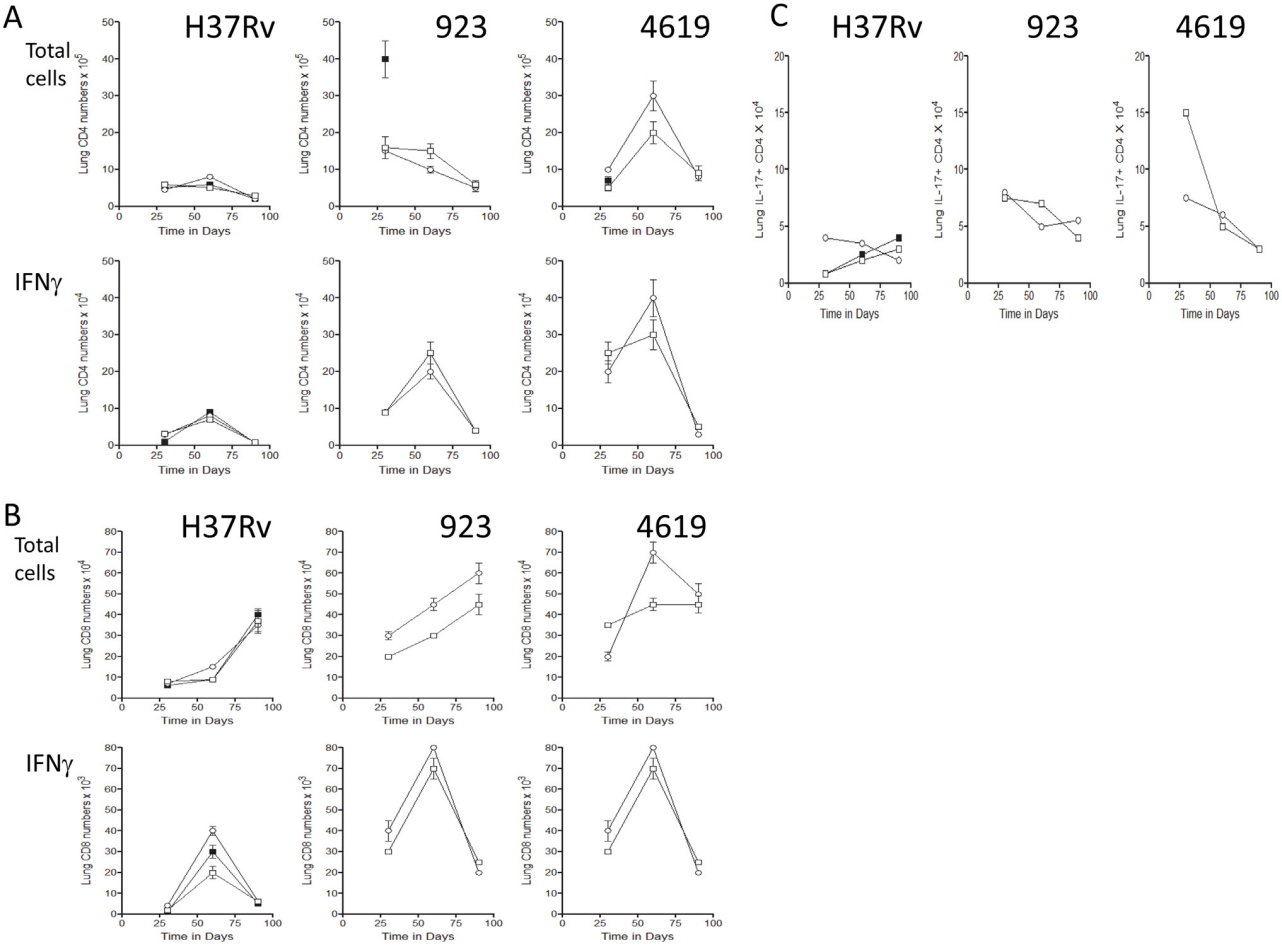
Flow cytometric analysis of the effector T cell response in the lungs infected with H37Rv, strain 923, or strain 4619. [A] Total CD4 T cell numbers and numbers of CD4 cells staining positive for IFNg. [B] Total CD8 T cell numbers and numbers of CD8 cells staining positive for IFNg. [C] Numbers of CD4 cells staining positive for IL-17A/F. Data is shown for saline controls [closed squares] and in mice previously vaccinated subcutaneously with BCG Pasteur [open circles] or rBCG Aeras 422 [open squares] [n = 4/5 mice ± SEM].

In addition to monitoring the bacterial load in the lungs of infected mice, we also gently digested and harvested T cells from the lung tissues for flow cytometric analysis [note: due to lung damage by the two clinical strains the control data was essentially unusable]. Total CD4 T cell influx was similar in each case (Fig 4A) and cells expressing IFN g represented about 10% of these, as anticipated based on earlier studies. No differences were seen between the two BCG vaccines used. Responses between strains were not linear however, with CD4 numbers increased in response to the two clinical isolates compared to H37Rv. A similar profile was seen in terms of the lung CD8 response, although here total numbers of cells were 90% lower (Fig 4B), as previously seen [8]. In addition, no major differences were seen in the numbers of CD4 cells expressing IL-17 (Fig 4C).

### Efficacy studies in the guinea pig model

**Fig 5:**
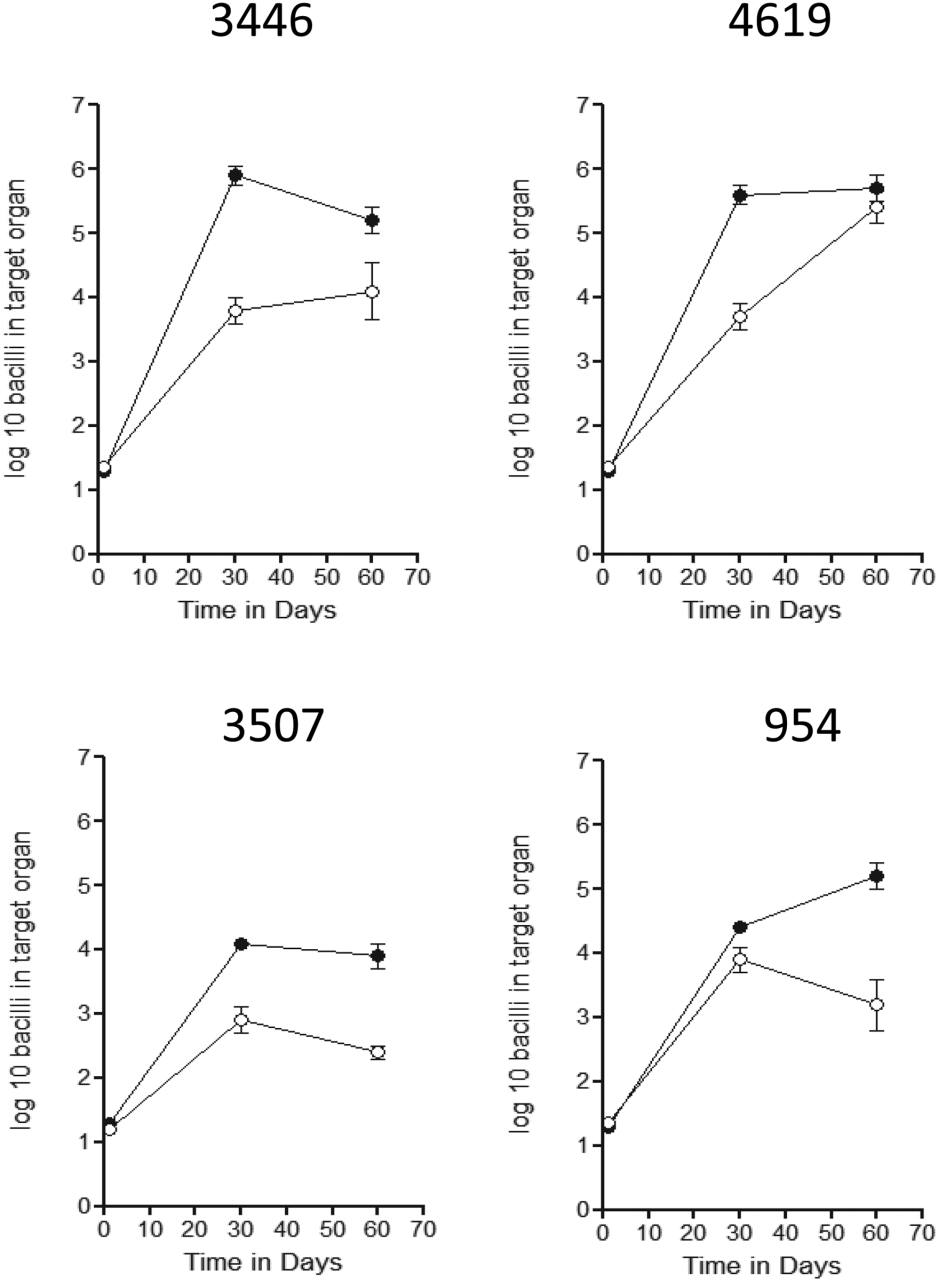
Course of infection following low dose aerosol exposure of guinea pigs to four Beijing strains, in saline controls [closed circles] or in animals previously vaccinated with BCG. Data is shown for groups of five animals ± SEM. Two strains [3507, 954] were contained and controlled by vaccination in these animals, whereas in the case of two others [3446, 4619] the bacterial load in the lungs was initially inhibited in the vaccinated animals on day-30, but this protection was lost by day-60.

**Fig 6:**
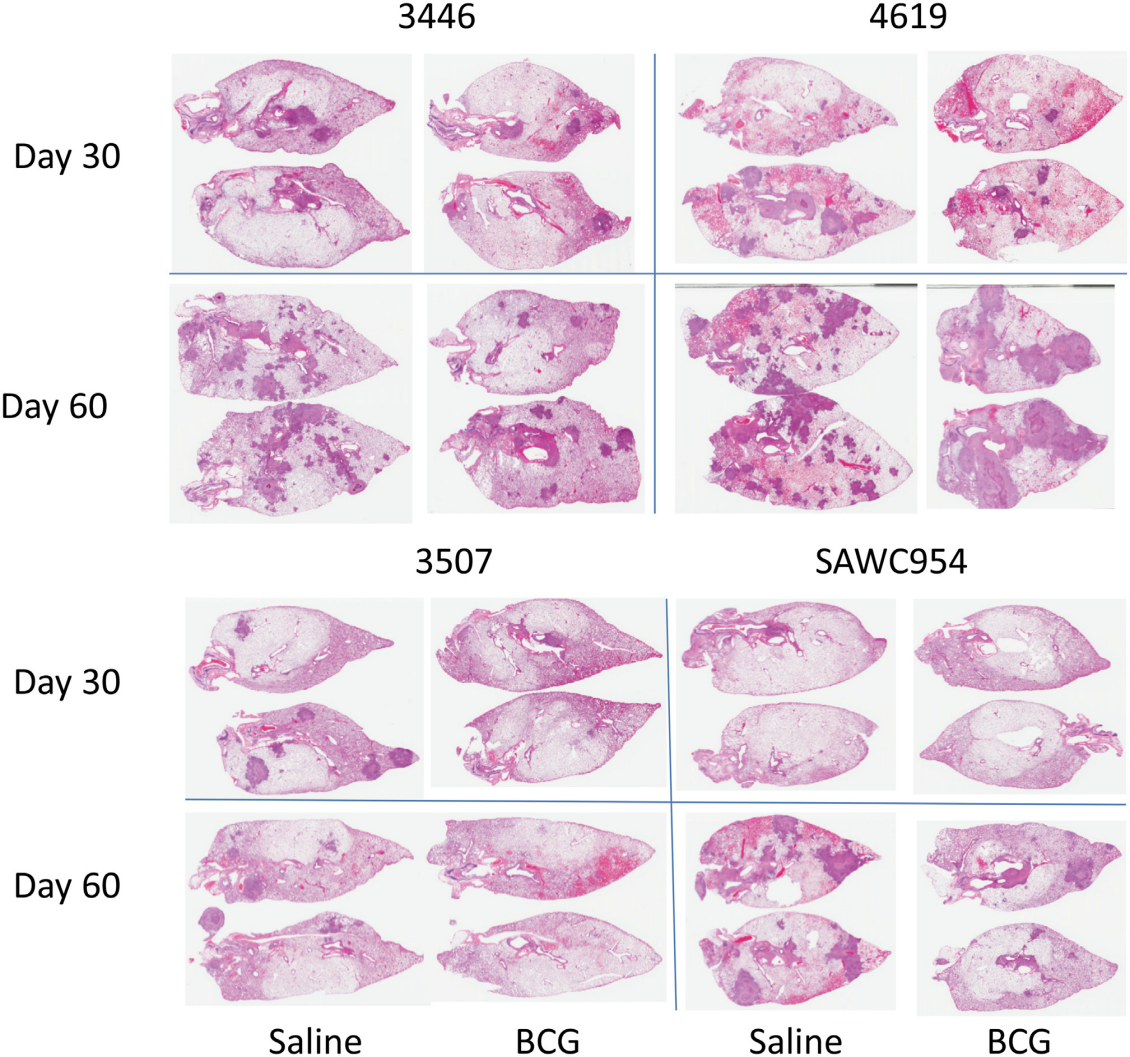
Representative histology images demonstrating overall disease burden in guinea pigs infected with four clinical strains and either sham-immunized or immunized with BCG. Pathology is compared at day 30 or day 60 of infection. Virulence, as indicated by overall disease severity and lesion extent, varied among the clinical isolates with 4619 causing the most extensive pathology. Protection provided by BCG immunization is proportionally evident, based on reduction in extent of pulmonary lesions.

In our next series of studies we tested BCG against several clinical isolates in the guinea pig low dose aerosol model. In a first experiment we tested BCG in animals subsequently infected with three Bay Area isolates [Beijings 3446, 4619, 3507] and a Western Cape strain [954]. In two cases [3507, 954] BCG behaved as it consistently does against laboratory strains, in controlling the challenge infection by day-30 and then slowly reducing the lung bacterial load thereafter (Fig 5). In the two other cases however, while BCG strongly inhibited the growth of 3446 and 4619 initially, the bacterial load continued to increase progressively, so that by day-60 it did not differ from the bacterial load in the unvaccinated control animals. These events were reflected by the lung pathology (Fig 6); one might note that while the actual number of lesions in the vaccinated animals infected with 4619 were fewer in number at day-60, the individual lesions were extremely large and highly necrotic.

**Fig 7:**
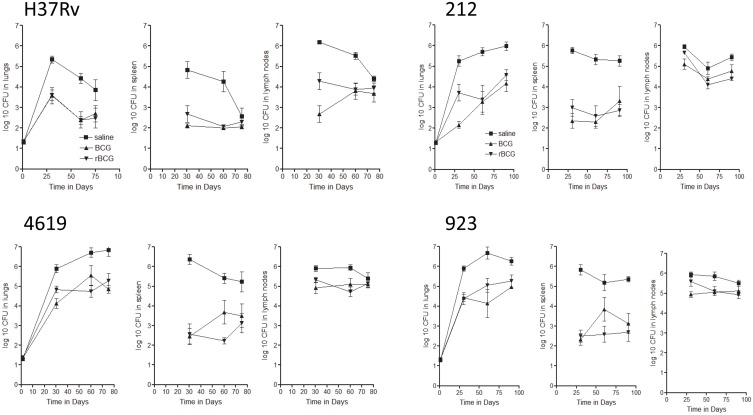
Course of infection in guinea pigs infected by low dose aerosol exposure to the laboratory strain H37Rv, the Beijing strains 212 and 4619, or the Haarlem strain 923. Data is shown for the three primary target organs, and based on five animals per group ±SEM. Animals were given saline subcutaneously [closed squares] or vaccinated with BCG [up triangle] or Aeras 422 [down triangle].

In a second set of studies in guinea pigs we compared H37Rv to the clinical isolates 4619, and 212 and 923 from the Western Cape, while also including rBCG Aeras-422. In these studies good protection was seen against H37Rv, but no difference was seen between animals given BCG or the rBCG in all three target organs (Fig 7). Better protection was seen here against 4619, although the bacterial load increased as before. BCG and the rBCG were both strongly protective against 212 and 923, although, again, the bacterial load in the lungs slowly increased by ~1-log in vaccinated animals from day-30 to day-90. Both vaccines strongly prevented initial dissemination of the infection to the spleen, but this was not observed in terms of carriage to the draining lymph nodes for all three clinical strains.

**Fig 8:**
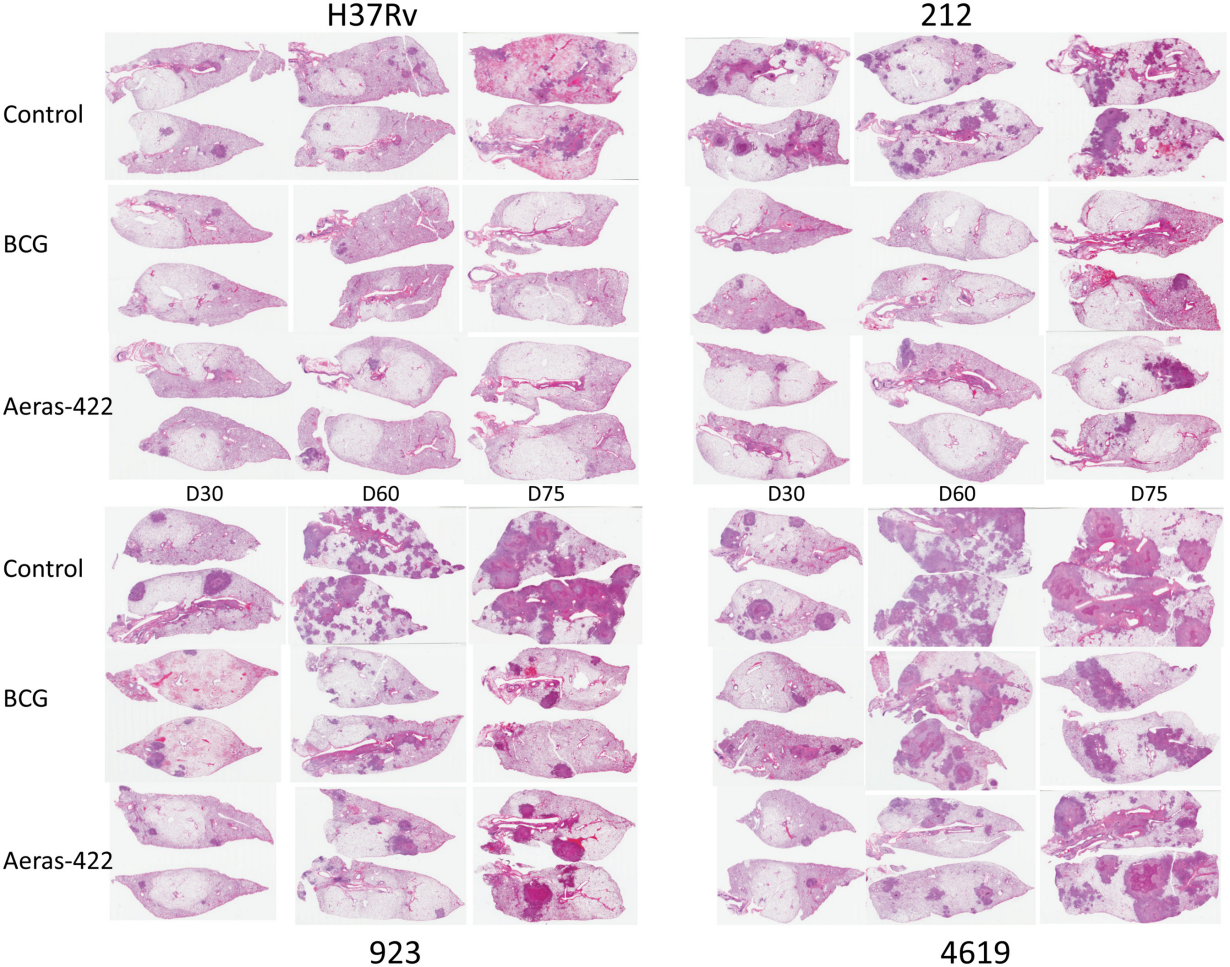
Representative histology images demonstrating overall disease burden in guinea pigs infected with H37Rv or three clinical strains, and either sham-immunized or immunized with BCG or Aeras 422. Pathology is compared at day 30, 60 or 75 of infection. Infection with clinical strains yielded more extensive pathology. Immunization with BCG or Aeras 422 led to reduced severity of disease overall in all cases, but no differences in protection afforded by BCG or Aeras 422 were evident.

Pathologic analysis (Fig 8) was consistent with these observations. Lesion development was almost completely inhibited in vaccinated animals infected with H37Rv. Very limited lesion development was seen in the 212 infection group, but a few large lesions developed by day-75. In the case of 923, very substantial lung damage developed in the control animals, which was inhibited by both BCG groups at day-60. Larger lesions become apparent however in the rBCG animals by day-75. Despite the better control of 4619 seen in this second study, lesion development was only partially reduced on day-60, but by day-75 large necrotic lesions had developed in both the BCG and rBCG groups.

### Long term survival studies in BCG vaccinated animals

**Fig 9:**
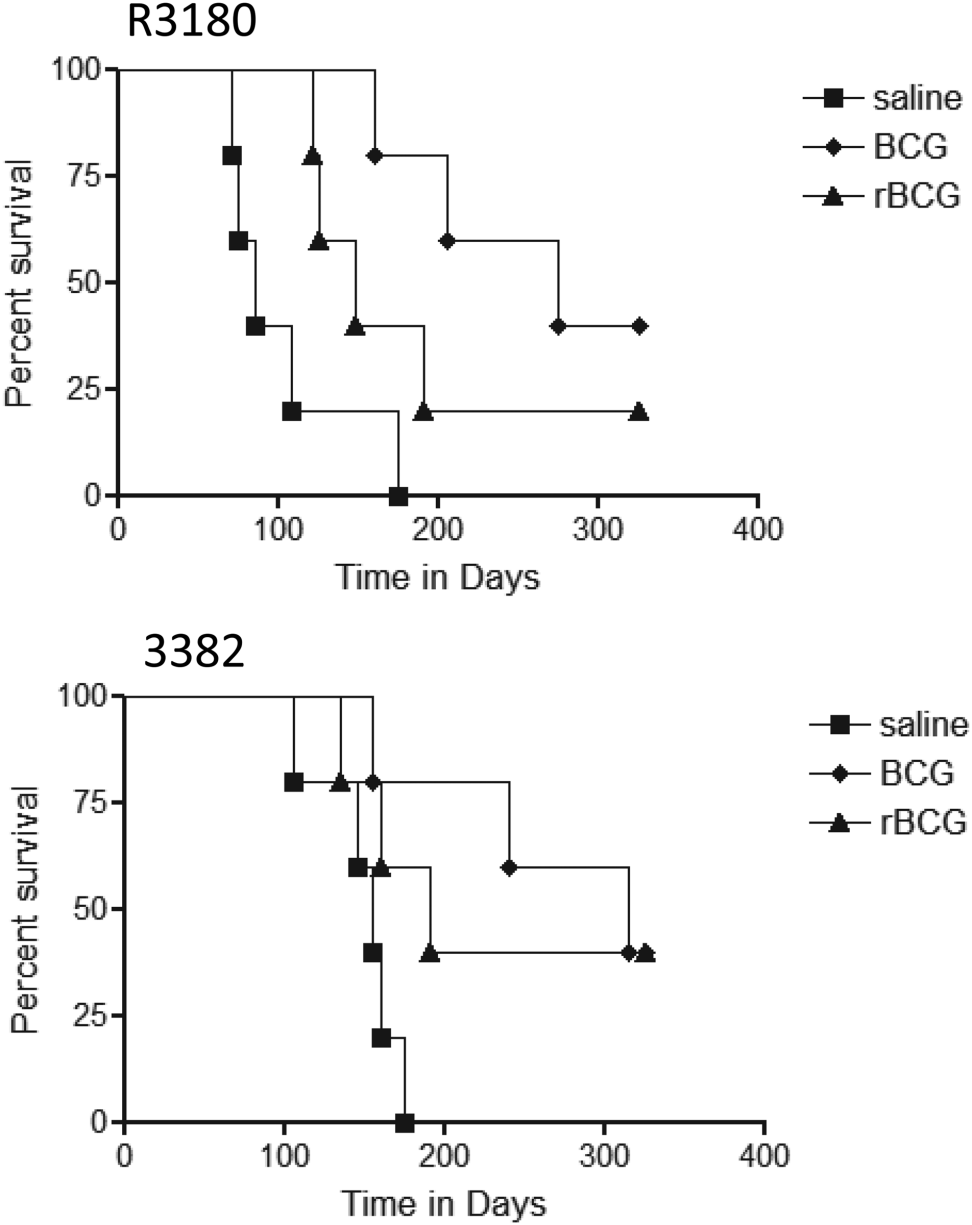
Kaplan Meier analysis of the survival of guinea pigs infected by low dose aerosol exposure to the Beijing strains R3180 or 3382. Data is based on 5–6 animals per group. BCG prolonged survival against strain 3180 [P = 0.006] whereas Aeras 422 did not [P = 0.056]. Similarly, BCG protected against strain 3382 [P = 0.014] whereas Aeras 422 did not [P = 0.07].

In several cases, BCG gave good protection over the short term, so we also addressed if this translated into long term protection, using a Kaplan Meier analysis. Guinea pigs were vaccinated with the two BCG vaccines, then infected with two Western Cape Beijing strains, 3382 and the rifampicin resistant strain 3180 (Fig 9). In the case of 3180 control animals died after ~90 days on average, and this was substantial extended in BCG vaccinated animals [P = 0.006] but not in animals given the rBCG [P = 0.056]. A similar result was seen using 3382, in which BCG extended survival significantly [P = 0.014] whereas rBCG did not [P = 0.07].

## Discussion

The results of this study show that animals vaccinated with BCG and then infected with newly emerging virulent isolates in general exhibit two profiles, in the context of how the course of the infection proceeds in the lungs. These two profiles are noted here, in previous studies in our laboratory [8,9,12], and by others [13], and can be observed in both the mouse and guinea pig models of tuberculosis.

The majority profile [with the caveat that we have only analyzed strains from two geographical regions—US outbreaks, and strains prevalent in the Western Cape] is where animals vaccinated with BCG gradually contain the infection by about thirty days, and the bacterial load remains at these lower levels thereafter or may even decline. The second profile is where BCG slows the infection at day-30 by 1-log or more, but the bacterial load continues to increase, to the extent that by day-60 it is essentially indistinguishable from the negative control animals. Our laboratory first observed this profile in mice using two extremely virulent US strains [HN878 and SA161], but we should also note that we have characterized other strains that are equally virulent which do not exhibit this, so virulence by itself is not an adequate explanation for this event. Analysis of the T cell response in the lungs in animals showing this loss of protection indicated that in mice the CD4 effector T cell response in the lungs at day-30 contracts and is then gradually replaced by CD4 Foxp3+ regulatory T cells [9], and similarly that in guinea pigs an increasingly strong Foxp3 mRNA message can be seen to develop in animals infected with these clinical strains [14].

Our overall studies to date indicate that clinical isolates from the Western Cape are very strongly inhibited by BCG. This is a consistent observation, and we have yet to see an isolate that is not—i.e. fits the second profile. And yet, at least some of these strains are extremely virulent in mice, causing rapid mortality [much faster in fact than the supposedly more susceptible guinea pig], but despite this both BCG and Aeras-422 were highly protective. In contrast, we observed a range of BCG efficacy against US outbreak strains, some are inhibited by BCG whereas others are not to any extent.

As we recently suggested [12,15] this may be less associated with virulence [strains like 923 and 212 are amongst the more virulent strains we have seen to date] and more a reflection of strain fitness. We previously noted that “US outbreak” strains, particularly those from the Bay Area [16] are circulating in populations where nutrition is adequate and the HIV rate is negligible, and it is from this overall collection that we have observed strains that are only transiently inhibited by BCG. In contrast, all the strains we have tested to date from the Western Cape region, in which malnutrition is an important factor and HIV rates are high [17–19], are all very strongly inhibited by BCG vaccination in our animal models. We propose therefore that BCG vaccination efficacy is primarily influenced by the fitness patterns of the strains against which it is tested, something not even considered to date given the reliance of the field on the laboratory strains used not only to test vaccines but which also dictates the current state of the “TB vaccine pipeline”.

For confidentiality reasons we do not know if our isolates from the Western Cape were from HIV-positive or HIV-negative individuals. We do know that some strains, such as strain 212, were associated with high transmission and could easily have passed through both populations. Degree of transmission is an important factor in overall bacterial fitness, and would be expected to be less in HIV-positive individual due to lack of cavities. Added to these factors, the immunogenicity of the strain is also a factor and we observed differences (Fig 4A) in the degree to which different strains induced IFNg^+^ CD4 T cells in the lungs. This in itself is also important when considered in concert with the degree of lung damage these strains can induce (Fig 3, for example); in other words, a strain can be low fitness but still immunogenic, leading to lung damage and transmission.

If our hypothesis is correct it has serious consequences for vaccine testing. In fact, this is already occurring, at least in the sense that negative results for MVA85A, which was very extensively tested and shown to be active in the mouse, guinea pig, and non-human primate animal models [7,20–23], have now led to some within our field advancing the concept that since MVA85A was tested extensively in animal models and the trial failed, then logically animal models are not predictive and should no longer be used. This is correct, in the sense that MVA85A was indeed extensively tested, but it was tested against the laboratory strains H37Rv or Erdman and never against any clinical strain, and certainly not against even a single clinical strain from the Western Cape region where the trial was actually conducted.

In our study here we consistently saw excellent protection, both with BCG and rBCG Aeras-422, against the Western Cape strains, consistent with earlier results with two further strains [12]. As we recently argued [12,15] if these strains are indeed of relatively low fitness then there is no way, both in modeling, or in reality in a clinical trial, that anything can be used that will boost BCG immunity to the extent that it can be statistically observed as an improvement on BCG alone. In other words, the fault lies not with MVA85A or with the animal models used to test it, but with the trial site itself in which the prevailing strains were sufficiently inhibited by BCG to the degree that this could not be improved upon. In the future we would suggest that the prevailing local strains in a clinical trial site area be collected and tested first, a relatively cheap process in small animal models, before a vastly more expensive trial is conducted. Furthermore, how any of the various new vaccine candidates in the current pipeline behave when animals are challenged with isolates of varying fitness should be immediately addressed.

A surprising aspect of these studies was that while rBCG Aeras-422 mostly gave similar protection compared to regular BCG, we found no evidence that it had any superior properties, which raises the question as to why [for a time at least] it featured in the TB vaccine “pipeline”. We mostly observed similar CFU patterns, found nothing to suggest that Aeras-422 elicited a stronger or faster effector T cell response, nor did we observe any evidence of changes or improvements in the lung pathologic appearance. Moreover, most of our studies consisted of conventional short term assays, but when we performed long term survival studies using two Western Cape isolates, Aeras-422 performed poorly in these assays and did not result in statistically significant improvement over non-vaccinated guinea pigs, whereas BCG improved survival in both cases. These data seem to suggest that while Aeras-422 generates adequate effector immunity, this immunity is not long lived. We have no data at present to explain this, but are currently looking at the possibility that this rBCG, given its over-expression of immunodominant antigens, may be pushing the acquired T cell response into exhaustion.

Modifying or boosting BCG seems a key tactic in vaccine development given the already wide coverage using this vaccine globally [24–27]. The genesis of the recent modifications to BCG that has resulted in new rBCG candidates was based firstly on the concept that better, longer lasting immunity would result when compared to conventional BCG, and secondly, in one example at least [28], that rBCG would be more effective against prevalent clinical isolate families, particularly Beijing isolates. Our results here support neither contention.
